# Further Study of the Polar Group’s Influence on the Antibacterial Activity of the 3-Substituted Quinuclidine Salts with Long Alkyl Chains

**DOI:** 10.3390/antibiotics12081231

**Published:** 2023-07-25

**Authors:** Renata Odžak, Doris Crnčević, Antonio Sabljić, Lucija Krce, Antonela Paladin, Ines Primožič, Matilda Šprung

**Affiliations:** 1 Department of Chemistry, Faculty of Science, University of Split, R. Bošković 33, 21000 Split, Croatia; rodzak@pmfst.hr (R.O.); dcrncevic@pmfst.hr (D.C.); asabljic1@pmfst.hr (A.S.); 2 Doctoral Study of Biophysics, Faculty of Science, University of Split, R. Bošković 33, 21000 Split, Croatia; 3 Department of Physics, Faculty of Science, University of Split, R. Bošković 33, 21000 Split, Croatia; lkrce@pmfst.hr; 4 Department of Biology, Faculty of Science, University of Split, R. Bošković 33, 21000 Split, Croatia; apaladin@pmfst.hr; 5 Department of Chemistry, Faculty of Science, University of Zagreb, Horvatovac 102a, 10000 Zagreb, Croatia; ines.primozic@chem.pmf.hr

**Keywords:** quaternary ammonium compounds, bacterial resistance, antibacterial activity, mechanism of action, cytotoxicity

## Abstract

Quaternary ammonium compounds (QACs) are among the most potent antimicrobial agents increasingly used by humans as disinfectants, antiseptics, surfactants, and biological dyes. As reports of bacterial co- and cross-resistance to QACs and their toxicity have emerged in recent years, new attempts are being made to develop *soft* QACs by introducing hydrolyzable groups that allow their controlled degradation. However, the development of such compounds has been hindered by the structural features that affect the bioactivity of QACs, one of them being polarity of the substituent near the quaternary center. To further investigate the influence of the polar group on the bioactivity of QACs, we synthesized 3-aminoquinuclidine salts for comparison with their structural analogues, 3-acetamidoquinuclidines. We found that the less polar amino-substituted compounds exhibited improved antibacterial activity over their more polar amide analogues. In addition to their better minimum inhibitory concentrations, the candidates were excellent at suppressing *Staphylococcus aureus* biofilm formation and killing bacteria almost immediately, as shown by the flow cytometry measurements. In addition, two candidates, namely QNH_2_-C_14_ and QNH_2_-C_16_, effectively suppressed bacterial growth even at concentrations below the MIC. QNH_2_-C_14_ was particularly effective at subinhibitory concentrations, inhibiting bacterial growth for up to 6 h. In addition, we found that the compounds targeted the bacterial membrane, leading to its perforation and subsequent cell death. Their low toxicity to human cells and low potential to develop bacterial resistance suggest that these compounds could serve as a basis for the development of new QACs.

## 1. Introduction

Quaternary ammonium compounds (QACs) have long been known as one of the most effective antimicrobial agents used by humans as disinfectants, antiseptics, surfactants, and biological dyes [1]. In addition to naturally occurring QACs, commercially available synthetic quaternary ammonium compounds are characterized by their potent antimicrobial activity, low production cost, and good stability [2]. Since their introduction at the beginning of the last century, this class of chemicals has been widely and increasingly used, raising concerns about their environmental and health impacts [3,4,5]. The occupational exposure of healthcare workers to QACs results in asthma [2] or toxicity to aquatic and terrestrial organisms [5,6]. Due to their physicochemical properties, QACs are readily adsorbed on various surfaces, which, together with their long degradation half-life, leads to accumulation in the environment at sublethal concentrations that promote toxicity and bacterial resistance [7].

The bacterial resistance to QACs is a serious problem that is gradually gaining attention, as the literature data indicate a higher rate of environmental isolates with acquired co- and cross-resistance genes [8], resulting in bacteria that are simultaneously resistant to QACs and common antibiotics [9]. Therefore, scientists are making efforts to decipher the resistance mechanisms and develop new QACs with high efficacy and a broad spectrum of activity.

Bacterial resistance to QACs is associated with changes in the lipid composition and the efflux system of the plasma membrane, which are related to *qac* genes [10,11]. Qac efflux pump expression is under the control of the transcriptional regulator QacR, which has been extensively studied and biochemically characterized [12,13,14]. These studies have shown that the recognition of QACs occurs through the electrostatic interaction between the positive quaternary center and the negatively charged residues at the bottom of the active site of the QacR. The location of the negatively charged residues is variable, resulting in structural plasticity that allows a large pool of QAC structures to be efficiently recognized and subsequently ejected from bacteria before they cause harmful cellular damage. Because QACs’ recognition is mediated by electrostatic interactions, Minbiole and co-workers have suggested that QACs bearing more than one positive quaternary center may be able to overcome QacR recognition, resulting in highly bioactive structures. Indeed, their extensive work on multiQACs has yielded structures with superior and broad activity [15,16,17]. To overcome bacterial resistance, the same research group has also proposed replacing the positive quaternary nitrogen with other atoms such as phosphonium or sulfur in mono- and multiQAC versions [18,19,20,21].

The other strategy to address the problems associated with these compounds is to develop *soft* QACs, i.e., variants with labile functional groups that allow spontaneous or enzyme-mediated degradation, limiting their half-life and shortening their residence time in the environment [22]. In this case, since bacteria are not subjected to selection pressure, *soft* QACs are not thought to induce bacterial resistance. The labile functional groups are usually esters or amides introduced near or at the junction of the quaternary nitrogen with the alkyl side chain(s), allowing efficient hydrolysis of the otherwise very stable structures [23]. The degradation products of *soft* QACs are nontoxic and therefore do not pose a threat to ecosystems, making them a good alternative to conventional QACs. However, some limitations related to the structure–activity relationship of *soft* QACs remain as obstacles for further study [24,25].

We have previously shown that naturally occurring bicyclic quinuclidine bearing a bridgehead nitrogen can be quaternized, resulting in potent QACs with a broad spectrum of antimicrobial activity with minimum inhibitory concentrations (MICs) in the range of nM units [26]. The introduction of an amide group into such a structure leads to *soft* QACs that are potentially degradable by trypsin but less active than originally thought [27]. In an attempt to further investigate the structure–activity relationship in the development of *soft* QACs, we synthesized three 3-aminoquinuclidine salts to compare with 3-acetamidoquinuclidine QACs, which did not exhibit antibacterial activity despite retaining the ubiquitous structural features, i.e., long alkyl chains at the quaternary center.

The effect of the polar substituent was investigated by determining the minimum inhibitory concentrations (MICs) for a panel of Gram-positive and Gram-negative bacteria. The candidates with the lowest MIC values were further evaluated for their potential to inhibit the bacterial biofilm formation. To determine the inhibitory potential of the candidates at levels below the MIC, time-resolved growth kinetics were performed, which allowed detailed inspection of the bacterial growth curves in the lag phase. Flow cytometry was used to perform time-kill experiments for the time-dependent quantification and differentiation of viable, damaged, and nonviable cells. Further characterization of the antibacterial candidates was performed by determining their potential to induce bacterial resistance and concluded with fluorescence microscopy measurements to investigate the mechanism of action. Given the potential application of these compounds as novel antibacterial agents, cytotoxicity was determined for healthy and tumorous human cell lines.

## 2. Results

### 2.1. Synthesis

**Scheme 1:**
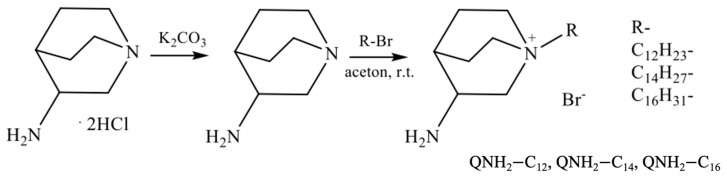
The reaction scheme shows the synthesis and structures of the obtained quaternary 3-aminoquinuclidine (QNH_2_) salts.

To investigate the influence of the polarity of the substituent on the antibacterial properties of QACs, we decided to compare two structural analogs that differed only by the substituent on the 3C-atom. 3-Amidoquinuclidine QACs were previously synthesized as described in [27], while 3-aminoquinuclidine QACs were obtained from commercial 3-aminoquinuclidine dihydrochloride as the starting material. After removal of the dihydrochloride, quaternization was carried out by the Menshutkin reaction using alkyl bromide reagents with the appropriate number of C-atoms (C12, C14, C16) (Scheme 1). The new derivatives were obtained in good yields (90–98%) and repeatedly crystallized to achieve the appropriate purity (Appendix A).

**Scheme 2:**
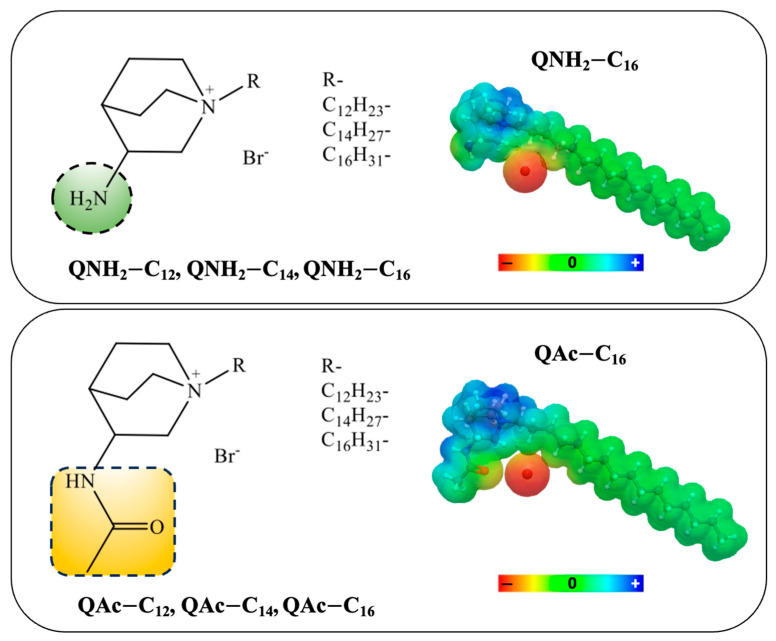
The structures of the quaternary 3-aminoquinuclidine (QNH_2_) and 3-acetamidoquinuclidine (QAc) salts with amino- and amide-substituents shown on the 3C-atom of the quinuclidine head. The structures of the corresponding derivatives with C16 alkyl chains with topological polar surface area indicating the polarity of the structures.

The QACs structures used in this study are shown in Scheme 2 highlighting the structural features and the differences between the two analogous groups. The structures exhibited different topological polar surface areas, with the amide-substituted salts having higher polarity due to the carbonyl oxygen at the 3C position of the quinuclidine head. In our previous study with pyridinium-4-adoxime, we similarly compared the structure–activity relationship of the two quaternary salts, namely the commercially available cetylpyridinium chloride (CPC) and its analog, which differed only by the substituent on the QACs backbone [27]. We found that the derivative with oxime functionalization at the backbone had reduced antibacterial activity compared with the otherwise active CPC without the oxime group, suggesting that the addition of polar groups at the quaternary backbone actually reduced the biological potential.

In addition to the polarity of the structure, the effect of the backbone rigidity on the antibacterial properties of the bis-QAC structures was also investigated [28]. It was found that the QACs with rigid core structures such as DABCO exhibited better antibacterial activity than the structural analogues with a less rigid piperazine backbone.

However, no further improvement in bioactivity was observed for the derivatives with amide functionalization in the alkyl side chains. A later study on the relationship between the rigidity and bioactivity of the bis-QACs structures apparently gave the opposite results [29], which may be due to the location of the quaternary nitrogen. Namely, in the first series, the quaternary centers were part of the ring structures, while in the other series, they were part of the two interconnected backbones.

Based on these and our results, we need to emphasize the importance of the QACs’ backbone structures in relation to the quaternary center. It seems that rigid cores with nitrogen as part of the ring structure lead to QACs with better antibacterial potential. However, the polarity of the substituent attached to the backbone and the position of the positive nitrogen could be equally important for the bioactivity.

### 2.2. Antibacterial Activity

The antibacterial activities of the 3-aminoquinuclidine precursor (QNH_2_) and the corresponding QACs (QNH_2_-C_12_, QNH_2_-C_14_, and QNH_2_-C_16_) were investigated by determining the minimum inhibitory concentrations (MICs) against six Gram-positive strains, namely, *Staphylococcus aureus* (ATCC25923, methicillin-resistant MRSA, and ATCC 33591), *Bacillus cereus* (ATCC 14579), *Listeria monocytogenes* (ATCC 7644), and *Enterococcus faecalis* (ATCC 29212), and three Gram-negative strains, *Escherichia coli* (ATCC 25922), *Salmonella enterica* (food isolate), and *Pseudomonas aeruginosa* (ATCC 27853). The influence of the polar group on the antibacterial properties of the QACs was resolved by comparing the obtained MIC values with the previously synthesized structurally similar quaternary salts of 3-acetamidoquinuclidine (QAc) [27] and two reference quaternary salts, namely benzalkonium bromide (benzyldimethyldodecylammonium bromide, BAB) and cetylpyridinium chloride (CPC).

In contrast to the 3-aminoquinuclidine (QNH_2_) and 3-acetamidoquinuclidine (QAc) precursors, which showed no antibacterial activity (Appendix B), the quaternary salts of the 3-aminoquinuclidine with long alkyl chains, QNH_2_-C_14_ and QNH_2_-C_16_, were modestly active against the Gram-positive strains (Table 1), except for the QNH_2_-C_12_.

Numerous literature data consistently confirm that QACs have better antibacterial potential against Gram-positive than Gram-negative bacteria [1]. This well-known observation can be explained by the differences in the membrane composition between Gram-negative and Gram-positive bacteria. Gram-positive bacteria have a thin cell envelope with an outer peptidoglycan layer enriched in teichoic acid [30]. Under physiological conditions, teichoic acid is negatively charged, which allows the electrostatic interaction with the positive N-atom of QACs. The proposed membranolytic mode of action involves a stepwise process consisting of the membrane integration of the QACs and the subsequent membrane perforation [15]. In contrast, Gram-negative bacteria have an outer phospholipid membrane in addition to their thin peptidoglycan layer, which provides further protection, resulting in higher MICs for QACs against these bacterial strains [30].

In our study, the activity against Gram-positive strains was also better, but as expected, the MIC values varied depending on the bacterial species and compound tested. For example, both the C12 derivatives of the 3-aminoquinuclidine and the 3-amidoquinuclidine QACs were not active in contrast to the C14 and C16 derivatives. As shown in Table 1, the MIC values of the C14 and C16 3-aminoquinuclidine salts were in the range from 12.5 μM to 100 μM against Gram-positive bacteria, with the exception of the multidrug-resistant *Staphylococcus aureus* strains (MIC ≥ 100 μM). Between these two candidates, the derivative with C16, namely QNH_2_-C_16_, was more effective against *S. aureus* ATCC 25923, *L. monocytogenes* ATCC 7644, and *E. faecalis* ATCC 29212 than the derivative with C14, which was consistent with the results of previous studies showing that derivatives with longer alkyl chains were more effective [1].

Interestingly, the QNH_2_-C_16_ derivative had excellent activity against *L. monocytogenes* ATCC 7644, similar to what we observed with 3-acetamidoquinuclidine QACs with a C16 chain [27]. The eradication of *L. monocytogenes* by conventional sanitization and disinfection methods has become increasingly difficult in recent years, as resistance to QACs has become more common [31,32,33]. *L. monocytogenes* is typically found in food processing environments, where surface sterilization techniques are frequently used, which means that the constant use of disinfectant in these environments could exert selection pressure on the bacteria leading to the development of resistance. For these reasons, a thorough investigation of the specific mechanism of action for *L. monocytogenes* is particularly important and is the focus of our further studies.

When comparing 3-aminoquinuclidine with 3-acetamidoquinuclidine QACs (Table 1), it is clear that 3-aminoquinuclidine derivatives exhibited superior activity against a greater number of Gram-positive bacteria tested, with low MICs against *S. aureus* ATCC 25923, *B. cereus* ATCC 14579, *L. monocytogenes* ATCC 7644, and *E. faecalis* ATCC 29212. Based on these results and our previous data, we once again showed that the addition of polar groups negatively affected the antibacterial properties of quaternary salts, as the finely tuned hydrophilic–hydrophobic character together with core rigidity is critical for antibacterial activity. Taken together, these results extend our knowledge of the structure–activity relationship of QACs and provide direction for the development of new effective and environmentally friendly disinfectants.

### 2.3. Inhibition of the Bacterial Biofilms

Bacterial biofilms are encapsulated collections of bacterial cells that have greater resistance to antibacterial reagents [34]. This is because the bacteria in the biofilm secrete extracellular polymeric substances that capture the bacterial cells, providing additional protection. A biofilm is a highly organized heterogeneous population that differs from bacteria in suspension by its cell density, lower oxygen content, and higher osmolarity. Due to their specific physiology, it is extremely difficult to eradicate bacterial biofilms, which is a serious problem in healthcare [35]. Bacterial biofilms colonize biotic and abiotic surfaces such as various medical devices, teeth, and wounds. Therefore, it is important to find antibacterial reagents that are effective not only against bacteria in suspension, but also against bacterial biofilms. This is even more important since biofilms are the predominant bacterial life form [36].

**Figure 1:**
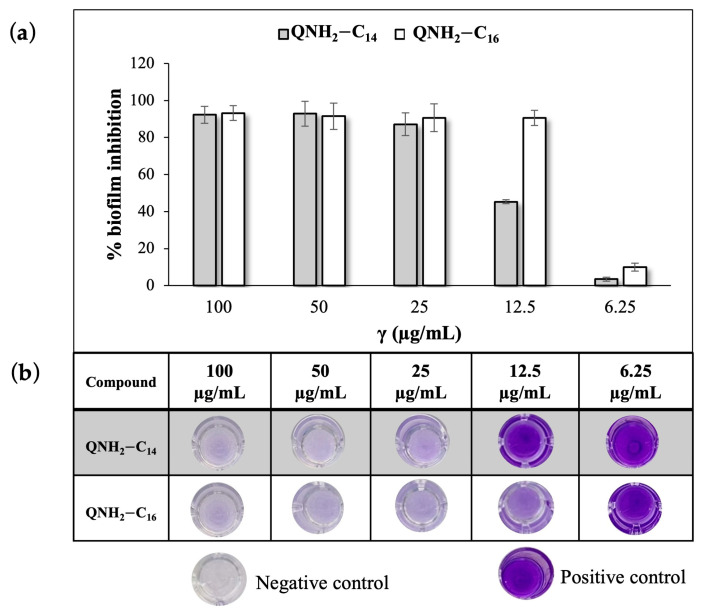
(**a**) Percentage (%) of the biofilm inhibition at a 100, 50, 25, 12.5, and 6.25 μg/mL concentration of QNH_2_-C_14_ and QNH_2_-C_16_ compared to the untreated *Staphylococcus aureus* ATCC 25923 biofilm (positive control). (**b**) The crystal violet staining of the *S. aureus* ATCC 25923 biofilms at the corresponding concentrations of the antibacterial agent.

The potential to inhibit *Staphylococcus aureus* ATCC25923 biofilm formation was determined for the selected candidates, namely, QNH_2_-C_14_ and QNH_2_-C_16_ (Figure 1).

As shown in Figure 1, the biofilm formation was almost completely inhibited at higher concentrations of both antibacterial agents (25–100 μg/mL). However, at a concentration of 12.5 μg/mL, the difference in antibiofilm activity became clear. QNH_2_-C_16_ inhibited the biofilm formation by more than 90%, whereas QNH_2_-C_14_ only inhibited 50% at 12.5 μg/mL. Since QACs are surface-active reagents, bacterial attachment to the wells of the polystyrene microtiter plates can be difficult, because the surface properties are altered by the coating [37]. Due to the higher surface activity of QNH_2_-C_16_, it was expected to effectively inhibit the biofilm formation. From these results, it can be concluded that both candidates QNH_2_-C_14_ and QNH_2_-C_16_ deserve further attention due to their excellent antibiofilm activities.

### 2.4. Time-Resolved Growth Analysis

**Figure 2:**
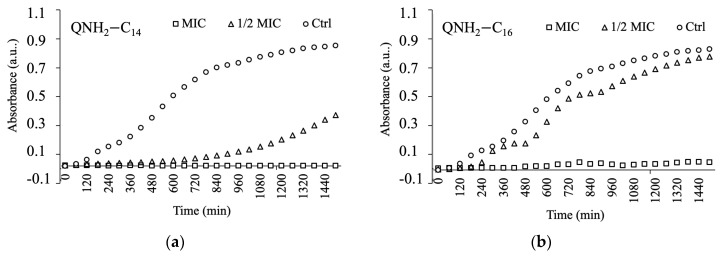
The growth kinetics of the untreated (Ctrl) and treated *Staphylococcus aureus* ATCC 25923 at MIC and ½ MIC concentration of the antibacterial agents, QNH_2_-C_14_ (**a**) and QNH_2_-C_16_ (**b**), during the 24 h of incubation.

The antibacterial activity of the quaternary 3-aminoquinuclidine salts was further investigated by following the dynamics of the bacterial growth in the presence of the antimicrobial reagent. The presence of the antimicrobial agent can alter the bacterial growth, revealing subtle differences in the efficacy of the antibacterial agents [38]. The growth dynamics of *Staphylococcus aureus* ATCC 25923 were followed in the presence of the antibacterial candidates at MIC and ½ MIC concentrations and compared to a control containing only untreated bacteria (Figure 2).

As shown in Figure 2a,b, the bacterial growth was completely suppressed at concentrations corresponding to the MICs. However, at ½ MIC, the different abilities of the candidates to suppress the bacterial growth were observed. Most striking were the differences in the duration of the lag phase. The lag phase is considered to be the phase of bacterial adaptation to new growth conditions that precedes the actual intensive cell division [3]. Since this phase is characterized by the adaptation to new environmental conditions rather than cell division, a detailed study of the growth curve during this phase may reveal subtle nuances in the efficacy of antibacterial agents. For example, QNH_2_-C_14_ was able to suppress the bacterial growth at ½ MIC during the first 360 min, whereas QNH_2_-C_16_ had a much shorter lag phase suppressing bacterial growth only during the first 200 min. In other words, it seems that QNH_2_-C_14_ is more efficient than QNH_2_-C_16_ in inhibiting bacteria, which is consistent with our previous results with 3-substituted quinuclidinol QAC bearing alkyl chains with 14 C atoms [26]. Other authors have also found better efficacy of QACs with shorter chains proposing that the reason could be the lower solubility of derivatives with chains ≥14 C-atoms [28].

In summary, this experiment demonstrated that QNH_2_-C_14_ and QNH_2_-C_16_ inhibited bacterial growth at concentrations below the MIC, albeit with different efficiencies, suggesting that these compounds might potentially be considered as new antibacterial candidates.

### 2.5. Potential of Bacterial Resistance Development

The development of bacterial resistance is an important parameter in the characterization of antimicrobial candidates. The major resistance mechanism against quaternary ammonium compounds is the plasma efflux system encoded by *qac* genes [1]. The efficacy of the efflux system in expelling QACs depends on the available intracellular ATP pool; so, in cases of a limited ATP supply, a decrease in MICs may indicate that the tested QACs are indeed substrates of the efflux system.

The protonophore carbonyl cyanide-3-chlorophenylhydrazone (CCCP) reduces the cellular ATP supply. The CCCP reagent has been shown to reduce the efflux activity of bacteria resistant to carbapenems [5] and, similarly, that of *Staphylococcus aureus* ATCC 33591, which is resistant to QACs [6,7]. Therefore, CCCP can be used to assess whether the synthesized QACs are substrates for Qac efflux pumps and the extent to which they induce bacterial resistance.

**Figure 3:**
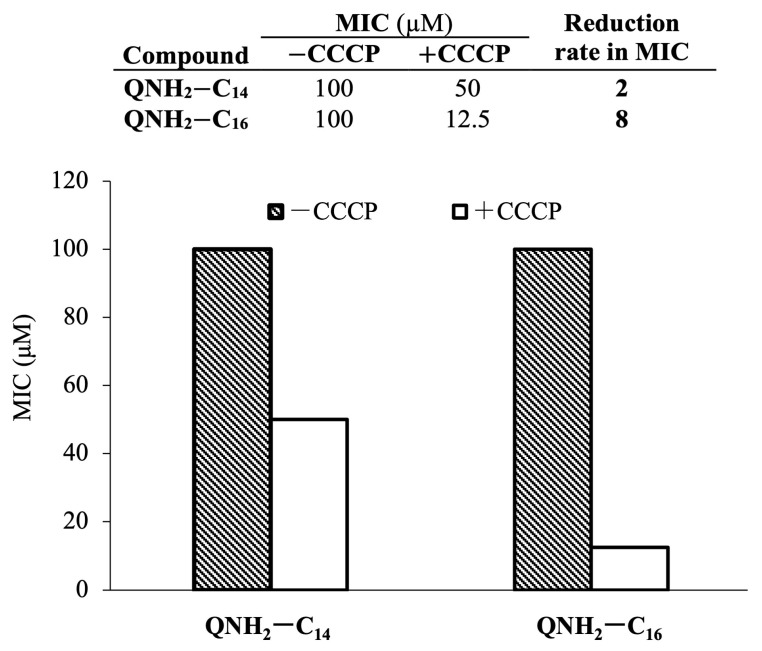
The reduction rate of the minimum inhibitory concentration for QNH_2_-C_14_ and QNH_2_-C_16_ without and with the presence of protonophore CCCP for *Staphylococcus aureus* ATTC 33591.

Figure 3 shows that QNH_2_-C_16_ was more likely to promote the development of bacterial resistance, due to the eightfold lower MIC in the presence of CCCP. On the other hand, the reduction in the MIC was twice as low with the QNH_2_-C_14_, indicating lower recognition of this compound by the bacterial intrinsic resistance system. It could be that the recognition of QACs is related not only to the electrostatic interaction but also to the length of the alkyl chain that must fit into the active site of the efflux pump or transcriptional regulator QacR. Another reason could be that QNH_2_-C_16_, due to its higher toxicity, triggers a rapid cellular response to survive and adapt to selection pressure.

### 2.6. Cell Viability in Relation to the Antibacterial Treatment Duration

Depending on how long it takes for an agent to kill bacteria, compounds displaying antimicrobial activity are generally divided into two broad groups. Bacteriostatic antimicrobials act mainly by inhibiting protein synthesis pathways, thereby keeping the bacterial population in the stationary growth phase and suppressing its division. In contrast, a bactericidal effect means that the population is killed during a 24 h incubation [39].

The flow cytometric detection of viable and nonviable bacteria is based on labeling the cells with a mixture of two fluorophores that can cross the bacterial membrane with different efficiencies and under different conditions of membrane integrity. Propidium iodide (PI) is a commonly used fluorescent dye that helps distinguish between live and dead bacteria. Due to the size of the molecule, PI can only penetrate damaged bacterial membranes. In contrast, the second fluorophore used in this experiment, thiazole orange (TO), served as a counter dye that stained the entire cell population.

**Figure 4:**
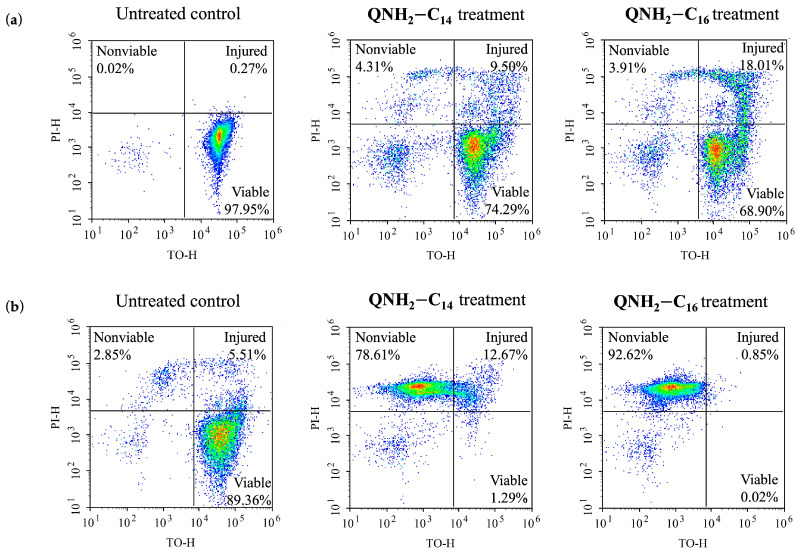
Flow cytometric evaluation of the viable and nonviable cells of *Staphylococcus aureus* ATCC 25923 after treatment with the minimum inhibitory concentration of QNH_2_-C_14_ and QNH_2_-C_16_ at the beginning of the experiment (**a**) and after four hours of treatment (**b**).

Since both candidates, QNH_2_-C_14_ and QNH_2_-C_16_, had low minimum inhibitory concentrations against *Staphylococcus aureus* ATCC 25923 (MIC = 25 µM), our aim was to investigate their time-dependent efficacy in annihilating the bacterial population. Figure 4 shows the flow cytometric detection of the untreated control sample compared to the sample treated with the MIC concentration of the candidate compounds at the start and after four hours of treatment.

In comparison with the untreated control, the measurements at the start of the treatment indicated an immediate toxic effect of both compounds towards the *S. aureus* population considering the spectral overlap of the TO and PI dyes. The prompt lethal action of the compounds can be seen in the relatively high ratio of injured cells, 9.50% and 18.01%, respectively, at the beginning of the treatment. Furthermore, the detection of the viable and nonviable cells after 4 h of treatment indicated the strong bactericidal action of the candidate compounds, pointing out that the QNH_2_-C_16_ was the most effective by killing over 90% of the *S. aureus* population. Considering that the intact membrane is impermeable to the fluorophore PI, the strong membranolytic activity of QNH_2_-C_16_ must be emphasized and further investigated.

### 2.7. Disruption of the Bacterial Cell Membrane

Since the proposed mode of action of QACs implies their membranolytic activity [15,40], the effect of the most potent compound on the bacterial membrane was investigated using optical fluorescence microscopy. For this purpose, immobilized *Listeria monocytogenes* ATCC 7644 cells were cultured in a sterile Petri dish in an appropriate nutrient broth and at optimal temperature. The undisturbed elongation and division of the cells before the treatment demonstrated the viability of the examined bacteria (Appendix C).

**Figure 5:**
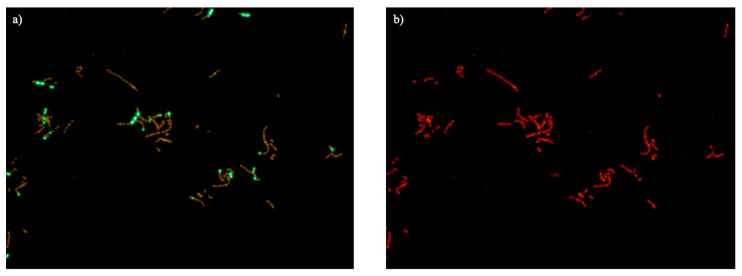
(**a**) Treated *Lysteria monocytogenes* ATCC 7644 cells stained with SYTO9; (**b**) the same treated cells stained with propidium iodide (PI).

Since QNH_2_-C_16_ was found to be the most potent candidate, it was selected to study its membranolytic activity. Therefore, *L. monocytogenes* cells were treated with the concentration of the desired compound corresponding to a 2xMIC. After three hours of treatment, the cells were stained with a mixture of green SYTO9 and red propidium iodide (PI) fluorescent dyes. These dyes are known as nucleic fluorophores and emit a fluorescent signal when intercalated with base pairs of DNA. While SYTO9 penetrates the membranes of living and dead cells, PI cannot diffuse through the conserved bacterial membrane and therefore stains only cells with damaged membranes [41]. Figure 5a shows the obtained fluorescence image of the SYTO9-stained cells, while (b) represents the same area stained with the propidium iodide (PI). Despite a few live cells labeled only with the SYTO9, which were therefore emitting an intense green fluorescence signal, the spectral overlap of the PI and SYTO9-stained cells in Figure 5a shows that most of the population had damaged membranes. This was also confirmed by the fluorescence image of the PI-stained cells pointing out the strong membranolytic activity of the QNH_2_-C_16_. It has been shown that QACs target the bacterial cell membrane and that this interaction occurs through the electrostatic attraction between the negatively charged groups in the bacterial membrane (e.g., teichoic acid) and the positive nitrogen of the QACs [15]. If the interaction is successful, hydrophobic parts of the QACs are incorporated into the membrane, leading to membrane destabilization and subsequent perforation, which results in bacterial death. Similarly, we have shown here that the treatment perforated the bacterial membrane, allowing the PI to enter the cell and bind to the DNA, resulting in red fluorescence.

### 2.8. Cytotoxicity

**Figure 6:**
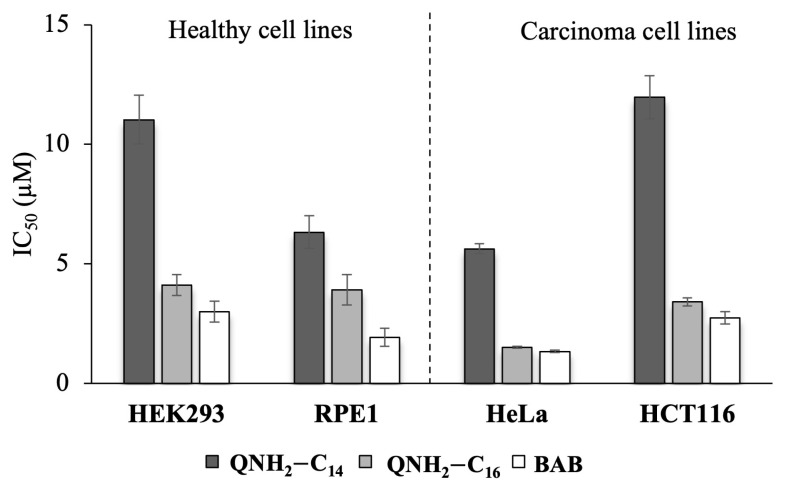
The toxicity of the QNH_2_-C_14_ and QNH_2_-C_16_ expressed as IC_50_ values for healthy (HEK, RPE1) and carcinoma (HeLa, HCT116) cell lines.

The toxicity of QACs is one of the major drawbacks of these compounds, which prevents their even wider use and application [42]. Therefore, it is not surprising that scientists in this field have recently been paying more attention to the development of environmentally friendly variants that are easily degradable and less toxic to terrestrial and aquatic organisms [22]. Furthermore, since QACs are commonly used as disinfectants and antiseptics, it is essential to ensure their safety for potential use in humans and animals. Therefore, the cytotoxicity of the selected candidates was investigated using healthy and carcinoma cell lines and compared with the commercial quaternary ammonium compound BAB. As shown in Figure 6, the two candidates had different toxicity profiles as indicated by the IC_50_ values. QNH_2_-C_14_ was less toxic to both the healthy and carcinoma cell lines, causing 50% dead cells only at higher concentrations compared to the untreated control. In contrast, the QNH_2_-C_16_ was more toxic and comparable in toxicity to the BAB for all cell lines. Our results suggest that both candidates may be viewed as new antibacterial candidates, given that they had better or comparable toxicity to the commercial BAB.

## 3. Materials and Methods

### 3.1. Synthesis

#### 3.1.1. Synthesis of (±)3-Aminoquinuclidine

Commercially available (±)3-aminoquinuclidine dihydrochloride (Sigma Aldrich, St. Louis, MI, USA) was treated with a saturated solution of potassium hydroxide to pH > 7. The mixture was subjected to chloroform extraction (10 × 5 mL), and the collected chloroform layers were dried on anhydrous potassium carbonate. The solvent was evaporated on the Büchi rotary evaporator to give (±)3-aminoquinuclidine that served as a reactant to produce appropriate quaternary ammonium salts.

#### 3.1.2. Synthesis of (±)3-Aminoquinuclidinium Quaternary Compounds

The quaternization reactions of (±)3-aminoquinuclidine were carried out with an equimolar amount of the appropriate quaternizing reagents: 1-bromododecane, 1-bromotetradecane, and 1-bromohexadecane (Alfa Aesar, Ward Hill, MA, USA). The reactions were carried out at room temperature in dry acetone with constant stirring over 2–3 days. The reactions were monitored by thin-layer chromatography using DC-Alufolien Aluminiumoxid 60 F254 (Merck, Readington Township, NJ, USA) with 9:1 chloroform–methanol as the eluent. The detection of spots was achieved by the reversible absorption of iodine. Repeated crystallization was required to attain the desired purity of the compounds. The melting points were determined in open capillaries using a Büchi B-540 apparatus and were uncorrected.

The ^1^H NMR spectra were recorded in a DMSO-d_6_ solution on a Bruker (Billerica, MA, USA) AV500 spectrometer (600 MHz) at room temperature. The chemical shifts are reported as δ values in ppm using TMS as an internal standard. Coupling constants (J) are given in Hz.

### 3.2. Broth Microdilution Assay

The antibacterial activity of the newly synthesized (±)3-aminoquinuclidine quaternary compounds was evaluated against Gram-positive (Staphylococcus aureus ATCC 25923, Staphylococcus aureus MRSA (clinical isolate), Staphylococcus aureus ATCC 33591, Bacillus cereus ATCC 14579, Listeria monocytogenes ATCC 7644, and Enterococcus faecalis ATCC 29212) and Gram-negative (Escherichia coli ATCC 25922, Salmonella enterica (food isolate), and Pseudomonas aeruginosa ATCC 27853) bacterial strains from BioGnost. The broth microdilution test was performed according to the Clinical and Laboratory Standard Institute’s “Methods for Dilution Antimicrobial Susceptibility Test for Bacteria That Grow Aerobically” [7]. Briefly, bacteria were inoculated into fresh Mueller–Hinton broth (MHB) (Biolife) with a sterile loop and cultured overnight at 37 °C with constant shaking at 220 rpm. The next day, the bacteria were diluted tenfold in fresh MHB and further incubated until the OD_600_ reached 0.3–0.5. The culture was diluted again to reach a cell concentration of 5 × 10^5^ CFU/mL. Then, using a multichannel pipette, 50 µL of the diluted cell culture was pipetted into previously prepared 96-well plates containing twofold serial dilutions of the compounds at a concentration range of 125 µM to 0.12 µM. The plates were incubated overnight at 37 °C, and the minimum inhibitory concentration (MIC) was determined at a concentration without visible bacterial growth. Subsequently, the visual determination of the MIC was confirmed by adding 20 µL of 2-(4-iodophenyl)-3-(4-nitrophenyl)-5-phenyltetrazolium chloride reagent (INT) (6 mg/mL), which turns purple in the presence of viable bacterial cells.

### 3.3. Biofilm Inhibition Assay

Minimum biofilm inhibitory concentrations (MBICs) were determined using the crystal violet staining method against *Staphylococcus aureus* ATCC 25923, which readily forms biofilms [43]. The overnight bacterial culture was diluted tenfold and allowed to continue growing until the exponential growth phase was reached. In parallel, 96-well microtiter plates were prepared by pipetting the antibacterial agents diluted in MHB at a concentration range of 100 to 12.5 µg/mL. Then, the exponentially grown culture (OD_600_ = 0.3–0.5) of *S. aureus* ATCC 25923 was diluted to a final concentration of 5 × 10^6^ CFU/mL, and 50 µL of this culture was added to the wells of the previously prepared microtiter plates. The plates were incubated for 48 h. After incubation, the broth over the biofilm was aspirated, and the plate with the biofilm was dried at 60 °C. After drying, 100 µL of a 1% crystal violet solution (CV) was added to the wells and incubated for one hour at room temperature. After staining, the solution of CV was aspirated, and the residual CV was rinsed with Milli-Q water. The CV stain was dissolved in 100 µL of 70% ethanol and the plates were further incubated for one hour. The absorbance was measured at 595 nm in an optical reader. The percentage of biofilm formed was calculated compared to the untreated control.

### 3.4. Time-Resolved Growth Analysis

The time-resolved growth analysis was performed in 96-well microtiter plates by incubating the antibacterial agent with Staphylococcus aureus ATCC 25923. Briefly, exponentially grown S. aureus ATCC 25923 (OD_600_ = 0.3–0.5) was diluted to a final concentration of 5 × 10^4^ CFU/mL in MHB. Then, 50 µL of this culture was added to the wells of the plate containing the MIC or ½ MIC of the compound to be tested. The plates were incubated overnight (24 h) at 37 °C in the microplate reader (Bio-Tek (Winooski, VT, USA) EL808) with shaking, recording the OD_600_ at 10 min intervals. The results obtained were compared to the untreated control and represent the mean of two independent experiments performed in triplicate.

### 3.5. Potential of Bacterial Resistance Development

The potential of the quaternary 3-aminoquinuclidine salts to induce bacterial resistance was determined in the presence of the ATP-synthesis inhibitor carbonyl cyanide-3-chlorophenylhydrazone (CCCP) (Sigma Aldrich) as previously described [44]. Prior to the experiment, the minimum inhibitory concentration of CCCP was determined against *Staphylococcus aureus* ATCC 33591 (MRSA). This strain was used because it contains efflux genes for QACs. On the day of the experiment, the overnight culture of *Staphylococcus aureus* ATCC 33591 was diluted in the Mueller–Hinton broth (MHB) and further incubated at 37 °C and 220 rpm until OD_600_ = 0.3–0.5. The culture was diluted to 5 × 10^5^ CFU/mL in MHB containing 10 µM CCCP. A total of 50 µL of the prepared cell suspension was added to a 96-well plate containing twofold serial dilutions of the tested compounds in MHB with CCCP at a final concentration of 10 µM. The plates were incubated overnight, and the MIC was determined the next day by visual inspection of the plates.

### 3.6. Time-Kill Kinetics Assay

Two overnight cultures of *Staphylococcus aureus* ATCC 25923 were grown at 37 °C and 220 rpm until the culture reached the pre-exponential growth phase. The cells were centrifuged at 4500× *g* for 10 min at room temperature. The supernatant was discarded and the cell pellet was resuspended in an equal volume of staining buffer (phosphate buffer, pH = 7.4, 1 mM EDTA, 0.1% Tween-20). The cell pellet from the second tube was resuspended in absolute ethanol, incubated for 15 min, centrifuged at the same speed, and finally resuspended in the staining buffer. Both prepared cultures were diluted in the desired volume of the staining buffer to a final cell concentration of 5 × 10^5^ CFU/mL and served as single-stained compensation controls along with unstained cells. For cell labeling and detection, a BD Biosciences (BD™ (Franklin Lakes, NJ, USA) Cell Viability) kit containing two fluorescent dyes (thiazole orange, TO, and propidium iodide, PI) was used according to the manufacturer’s instructions. The suspension of the cells in staining buffer (5 × 10^5^ CFU/mL) was pipetted into the 96-well plate containing the MIC and 2xMIC concentrations of the tested compounds dissolved in sterile staining buffer. Each plate was incubated at 37 °C for 24 h. At the desired time intervals (0, 2, 4, 6, 8, 10, 12, and 24 h), the treated cells were stained with a mixture of TO and PI, and the cell viability was measured using a NovoCyte Advanteon flow cytometer.

### 3.7. Optical Fluorescence Microscopy Measurements

Sterile Petri dishes (WPI, Sarasota, FL, USA) were coated with Cell-Tak (Corning, New York, NY, USA) solution and incubated for half an hour. After incubation, the dishes were rinsed six times with Mili-Q water and air-dried in the laminar flow hood. An aliquot of the pre-exponentially grown culture of *Listeria monocytogenes* ATCC 7644 was transferred to the coated Petri dish and incubated for 10 min. The unbound cells were rinsed thoroughly, taking care not to dry out the sample. The remaining immobilized cells were incubated in nutrient broth medium at 35 °C for an additional hour. To demonstrate the elongation and division of the attached cells, bright field images were taken at the beginning and end of the incubation. After confirming the viability of the immobilized bacteria, the cells were treated with an appropriate volume of QNH_2_-C_16_ equivalent to 2xMIC. Three hours after the treatment, the QNH_2_-C_16_ solution was replaced with sterile physiological saline. The cells were stained in the dark with a mixture of SYTO9 and propidium iodide (PI) nucleic fluorophores from the LIVE/DEAD™ *Bac*Light™ Bacterial Viability Kit (Thermofischer Scientific, Waltham, MA, USA) at a final dye concentration of 1.5 µL/mL. Fluorescence images were taken half an hour after staining.

### 3.8. Cytotoxicity

The cytotoxicity of the quaternary 3-aminoquinuclidine salts was tested on healthy (HEK293 and RPE1) and tumor cell lines (HeLa and HCT116). Fully confluent plates containing human cells were cultured in DMEM medium at 37 °C in a humidified atmosphere containing 5% CO_2_. On the day of the experiment, the cells were detached from the plate surface with trypsin and resuspended in DMEM medium by gentle pipetting. The cell concentration was determined using a handheld cell counter (Scepter, Merck) and adjusted to 1 × 10^5^ cells/mL using DMEM. The cytotoxicity was determined in the microtiter plates containing a serial dilution of the tested compounds in a concentration range of 250 µM to 0.25 µM and 50 µL of the appropriate cells (5000 cells/well). After 48 h of incubation, the percentage of live cells was determined by adding 20 µL of the reagent MTS (CellTiter 96^®^ Aqueous One Solution Cell Proliferation Assay, Promega, Madison, WI, USA) and recording the absorbance at 490 nm. The IC_50_ values were calculated using GraFit 6.0 software and are presented as the mean value of three independent experiments performed in duplicates.

## 4. Conclusions

Quaternary ammonium compounds (QACs) are among the most effective antimicrobial agents and are the main components of many disinfectants and antiseptics. Due to their widespread use, especially in recent years, concerns have been raised about the increasing bacterial resistance and toxicity to terrestrial and aquatic organisms. Therefore, considerable efforts have been made to develop new and effective variants of QACs that degrade spontaneously or are enzymatically releasing products that do not pose a threat to ecosystems. Such *soft* QACs have inherent amide- or ester-modified substituents that can be readily hydrolyzed to nontoxic products. Based on this idea, we designed and synthesized several series of 3-amidoquinuclidine QACs in our previous study. These series of QACs showed that the antibacterial potential depended not only on the backbone rigidity but also on other structural elements such as the polarity of the substituents. Here, to further investigate this hypothesis, we synthesized quinuclidine QACs with less polar amine functionalization. We found that the amine derivatives with longer chains, e.g., QNH_2_-C_14_ and QNH_2_-C_16_, exhibited good antibacterial potential and effectively killed bacteria immediately after treatment. One of the candidates, namely QNH_2_-C_14_, was able to effectively suppress the bacterial growth for up to 6 h even at concentrations below the MIC. The fluorescent labeling of the treated and untreated bacterial cells showed that these compounds targeted the cell membrane, as evidenced by a high number of PI-positive cells after treatment. The identified candidates have a low toxicity to healthy and carcinoma cell lines, comparable to the commercial quaternary salt BAB and can therefore be considered safe for usage in humans. In conclusion, amine-containing QACs have better antibacterial properties and are generally more effective than amide analogs, suggesting that this structural feature needs to be considered in the future development of new QACs. Our results highlight the importance of substituents on the QAC backbone and suggest that this feature is essential for their antibacterial activity.

## Figures and Tables

**Table 1:** Antibacterial activity (MIC/µM) of the new 3-aminoquinuclidine QACs in comparison with their analogs and commercial QACs standards (BAB and CPC).

			MIC (µM)							
Species			Compound							
***Gram-positive***			**QNH_2_-C_12_**	**QNH_2_-C_14_**	**QNH_2_-C_16_**	QAc-C_12_ *	QAc-C_14_ *	QAc-C_16_ *	**BAB**	**CPC**
		**Strain Origin**	***M*_r_ = 375.4**	***M*_r_ = 403.5**	***M*_r_ = 431.6**	***M*_r_ = 417.5**	***M*_r_ = 445.5**	***M*_r_ = 473.6**	***M*_r_ = 384.4**	***M*_r_ = 340**
*Staphylococcus aureus*		ATCC 25923	100	**25**	**25**	>125	>125	>125	10.00	4.00
*Staphylococcus aureus*		Clinical/MRSA	>100	>100	>100	>125	>125	>125	25.00	8.00
*Staphylococcus aureus*		ATCC 33591	>100	100	100	>125	>125	>125	25.00	6.00
*Bacillus cereus*		ATCC 14579	100	**25**	**12.5**	>125	>125	>125	25.00	16.00
*Listeria monocytogenes*		ATCC 7644	100	**50**	**12.5**	>125	**63**	**8**	10.00	8.00
*Enterococcus faecalis*		ATCC 29212	100	100	**50**	>125	**63**	**31**	15.00	8.00
***Gram-negative***										
*Escherichia coli*		ATCC 25922	>100	100	100	>125	>125	>125	63.00	16.00
*Salmonella enterica*		food isolate	>100	>100	>100	>125	>125	>125	50.00	63.00
*Pseudomonas aeruginosa*		ATCC 27853	>100	>100	>100	>125	>125	>125	>125	250

* MIC values were determined in [1].

**Figure A1:**
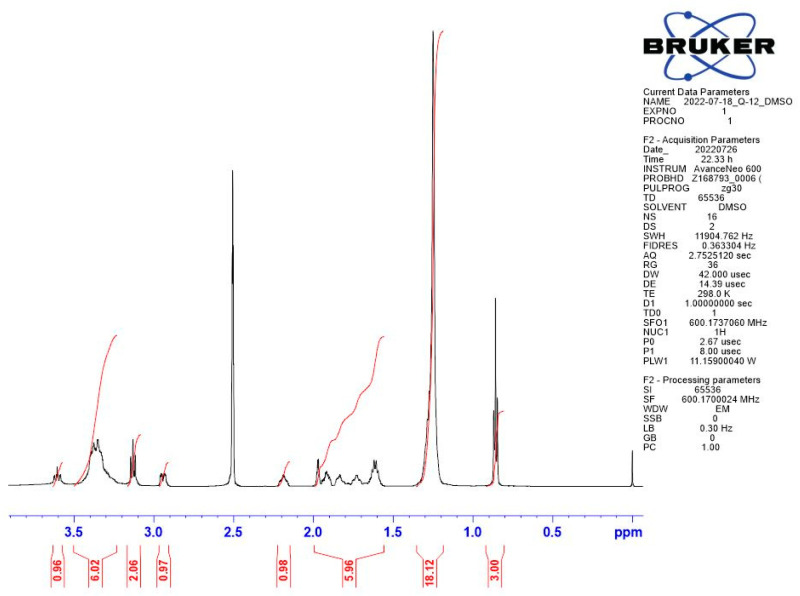
**^1^H NMR** (600 MHz, DMSO-d_6_) *δ*/ppm: 0.86 (*t*, *J* = 6.9 Hz, 3H, H12′); 1.18–1.34 (m, 18H, H3′–H11′); 1.56–1.99 (m, 6H, H5, H8, H2′); 2.15–2.22 (m, 1H, H5); 2.91–2.97 (m, 1H H4); 3.13 (*t*, *J* = 8.5 Hz, 2H, H1′); 3.23–3.50 (m, 6H, H6, H7, H2); 3.57–3.63 (m, H3).

**Figure A2:**
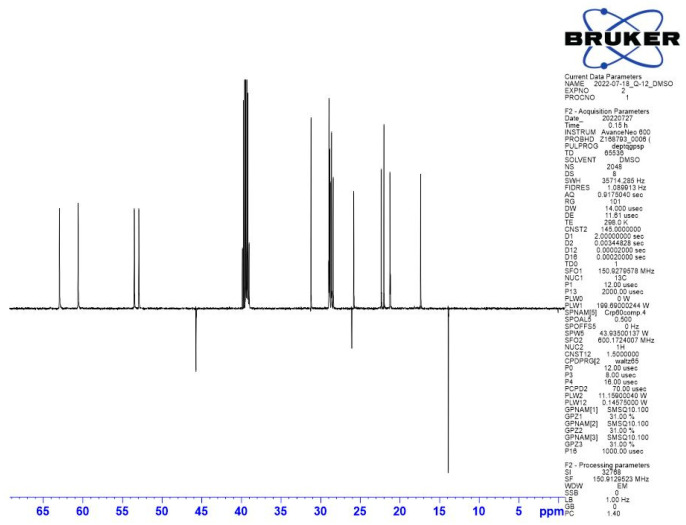
**^13^C NMR** (150 MHz, DMSO-d_6_) δ/ppm: 13.89 (C12′); 17.38, 21.24, 22.01, 22.40, 25.84, 28.37–29.03, 31.21 (C2′–C11′, C5, C8); 26.01 (C4); 45.71 (C3); 52.94, 53.54 (C6, C7); 60.63, 62.94 (C2, C1′).

**Figure A3:**
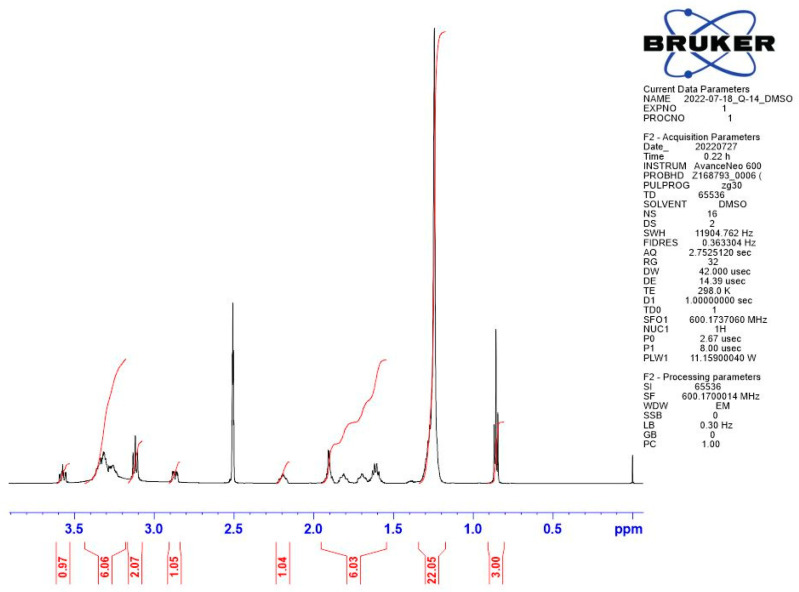
**^1^H NMR** (600 MHz, DMSO-d_6_) δ/ppm: 0,86 (*t*, *J* = 6.9 Hz, 3H, H14′); 1.18–1.36 (m, 22H, H3′–H13′); 1.56–1.95 (m, 6H, H5, H8, H2′); 2.16–2.23 (m, 1H, H5); 2.84–2.90 (m, H4); 3,12 (*t*, *J* = 8.5 Hz, 2H, H1′); 3.22–3.45 (m, 6H, H6, H7, H2); 3.55–3.61 (m, H3).

**Figure A4:**
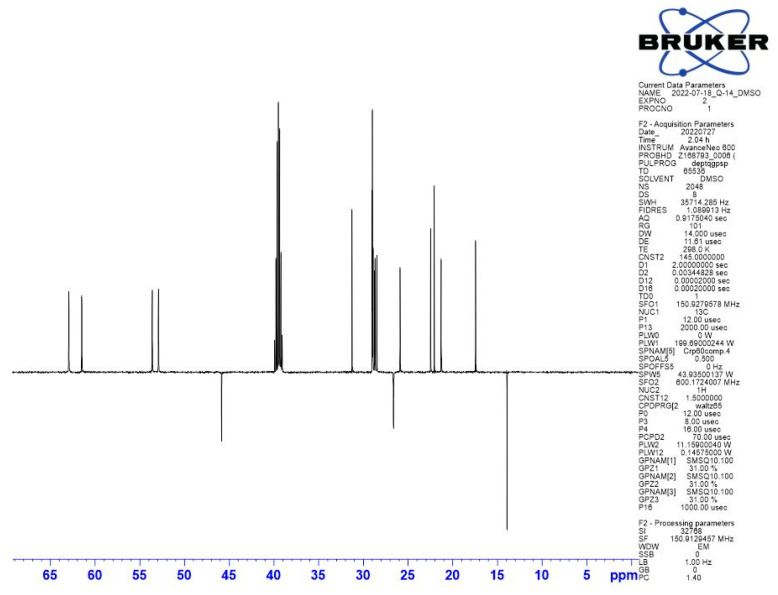
**^13^C NMR** (150 MHz, DMSO-d_6_) δ/ppm: 13.94 (C14′); 17.42, 21.27, 22.04, 22.46, 25.87, 28.46–29.01, 31.25 (C2′–C13′, C5, C8); 26.61 (C4); 45.85 (C3); 52.91, 53.61 (C6, C7); 61.50, 62.94 (C2, C1′).

**Figure A5:**
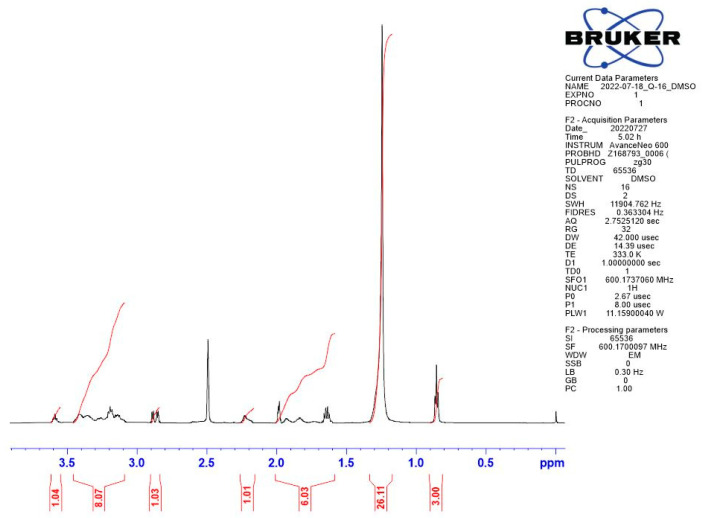
**^1^H NMR** (600 MHz, DMSO-d_6_) δ/ppm: 0.86 (*t*, *J* = 6.9 Hz, 3H, H16′); 1.16–1.38 (m, 26H, H3′–H15′); 1.57–2.01 (m, 6H, H5, H8, H2′); 2.16–2.26 (m, 1H, H5); 2.84–2.90 (m, H4); 3.09–3.45 (m, 8H, H6, H7, H2, H1′); 3.55–3.61 (m, H3).

**Figure A6:**
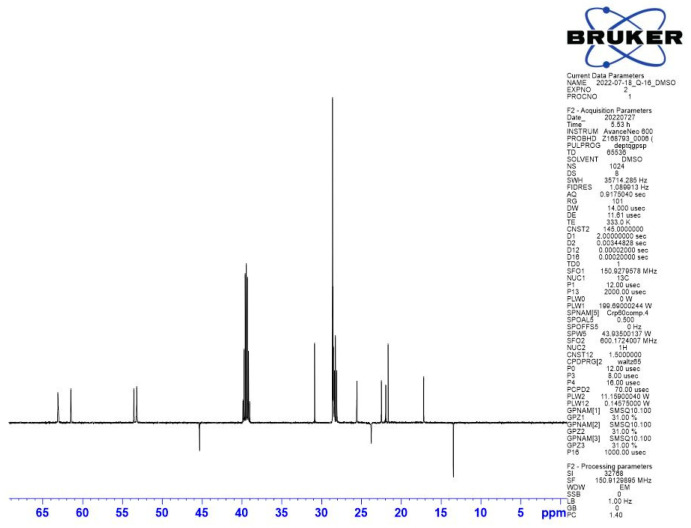
**^13^C NMR** (150 MHz, DMSO-d_6_) δ/ppm: 13.47 (C16′); 17.21, 21.69, 21.97, 25.62, 28.17–28.75, 30.97 (C2′–C15′, C5, C8); 23.81 (C4); 45.35 (C3); 53.11, 53.61 (C6, C7); 61.50, 63.19 (C2, C1′).

**Figure A7:**
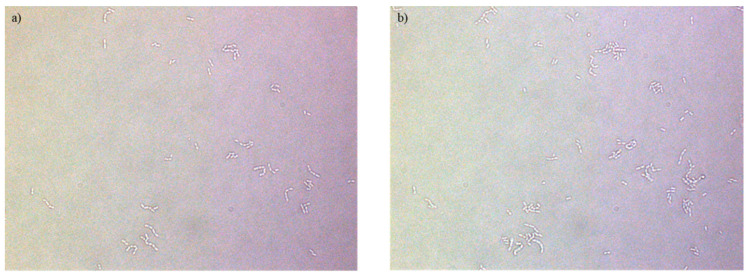
Bright-field image of (**a**) the immobilized *Listeria monocytogenes* ATCC 7644 cells; (**b**) the elongation and division of the same cells after one hour of incubation in the nutrient broth.

## Data Availability

The data presented in this study are available on request from the corresponding author.

## Appendix A

*N*-dodecyl-3-aminoquinuclidinium bromide (QNH_2_-C_12_): *η* = 90%, *m.p.* 162–164 °C.

*N*-tetradecyl-3-aminoquinuclidinium bromide (QNH2-C14): *η* = 98%, *m.p.* 1695–198 °C.

*N*-hexadecyl-3-aminoquinuclidinium bromide (QNH2-C16): *η* = 97%, *m.p.* 206–208 °C.

## Appendix B

**Table A1:** The minimum inhibitory concentration (MIC/µM) of the 3-aminoquinuclidine and 3-amidoquinuclidine QAC precursors.

Species	Strain Origin	(MIC)/µM	
**Gram-positive**		**QNH_2_**	**QAc**
*Staphylococcus aureus*	ATCC 25923	>100	>100
*Staphylococcus aureus*	Clinical/MRSA	>100	>100
*Staphylococcus aureus*	ATCC 33591	>100	>100
*Bacillus cereus*	ATCC 14579	>100	>100
*Listeria monocytogenes*	ATCC 7644	>100	>100
*Enterococcus faecalis*	ATCC 29212	>100	>100
**Gram-negative**			
*Escherichia coli*	ATCC 25922	>100	>100
*Salmonella enterica*	food isolate	>100	>100
*Pseudomonas aeruginosa*	ATCC 27853	>100	>100
